# Enteric neurosphere cells injected into rectal submucosa might migrate caudorostrally to reconstitute enteric ganglia along the entire length of postnatal colon

**DOI:** 10.1186/s13287-022-03187-2

**Published:** 2022-10-09

**Authors:** Jeng-Chang Chen

**Affiliations:** Department of Surgery, Chang Gung Children’s Hospital, College of Medicine, Chang Gung University, 5, Fu-Shin Street, Kweishan, Taoyuan, 333 Taiwan

**Keywords:** Enteric neurosphere, Enteric neural stem cell, Ganglion reconstitution, Rectal submucosal injection, Transplantation

## Abstract

**Background:**

In enteric neural stem cell (ENSC) therapy for enteric neuropathy, the gut is ostensibly accessible via laparotomy, laparoscopy or endoscopy, whereas its elongated configuration and multilayered structures substantially complicate the targeting of ENSC delivery. This study aimed to evaluate the feasibility of ENSC delivery via trans-anal rectal submucosal injection.

**Methods:**

ENSC transplantation was conducted in an immunologically compatible model of FVB/NCrl-Tg(Pgk1-EGFP)01Narl into FVB/N murine strain combination. Enteric neurospheres were mass-produced by the cultivation of dispersed enterocytes harvested from gestational day 14 FVB/NCrl-Tg(Pgk1-EGFP)01Narl murine fetuses. Dissociated neurosphere cells were injected into rectal submucosa of adult FVB/N mice after artificial prolapse of rectal mucosa. Ganglion reconstitution in recipients’ colon was examined by immunohistochemcal and immunofluorescence staining.

**Results:**

Cell spreading and ganglion assembly in recipients’ colorectum were examined one week after transplantation. Donor ENSCs migrated rostrally within the colonic wall to intermuscularly repopulate the neighboring colorectum and assemble myenteric ganglia. It contributed to a chimeric state of myenteric plexuses with donor-origin ganglia of 41.2–67.5%. Two months later, transplanted ENSCs had undergone long-distance caudorostral migration almost up to the cecum to reconstitute myenteric and submucosal ganglia along the entire length of the colon.

**Conclusion:**

This proof-of-principle study provided a viable justification for minimally invasive rectal ENSC transplantation to create long-term and long-range reconstitution of enteric ganglia. It opens up the new approach to ENSC delivery in laboratory animals and casts light on the feasibility of replacing damaged or replenishing missing enteric neurons by trans-anal rectal ENSC transplantation.

**Supplementary Information:**

The online version contains supplementary material available at 10.1186/s13287-022-03187-2.

## Introduction

Over the past two decades, enteric neural stem cell (ENSC) therapy for enteric neuropathy has fascinated the communities of gastroenterology, surgery and regeneration medicine. Arguably, ENSC therapy for Hirschsprung’s disease (HSCR) exemplifies this promise and has sparked extensive explorations in animal models [1–3]. To take ENSC therapy from experimental concept to clinical reality relies upon the procurement of sufficient ENSCs and an effective route for ENSC delivery [4–6]. The enteric nervous system (ENS) consists of myenteric and submucosal plexuses, embedded in gut wall. This poses a considerable challenge to not only ENSC isolation from the gut but also ENSC delivery into the proper strata of the multilayered gut wall. Following remarkable advances in the knowledge and technique of ENSC propagation [5–8], there has been a growing trend toward isolating ENSCs in the form of neurospheres through the cultivation of heterogenous enterocytes [7, 8]. As to cell delivery in ENSC therapeutics, the gut is ostensibly accessible via laparotomy, laparoscopy or endoscopy, whereas its elongated configuration and multilayered structures substantially complicate the targeting of ENSC delivery and the engraftment assessment after transplantation. In preclinical murine studies, cell injection was liable to the penetration of injection needles through the extremely thin gut wall [6, 9], let alone histologically stratum-specific cell delivery. Of note, the engraftment analyses after ENSC transplantation were mainly confined to the focal spreading of donor cells in the vicinity of injection site [2, 10–13], but fell short of thorough histological examinations for the long-range cell distribution to justify their clinical potential. This proof-of-principle study aimed to explore the feasibility of trans-anal approach to ENSC delivery in the murine model. It demonstrated that ENSC transplantation into rectal submucosa might cause long-range and long-term reconstitution of enteric ganglia.

## Methods

### Mice

Inbred FVB/N(H-2^q^) and transgenic FVB/NCrl-Tg(Pgk1-EGFP)01Narl mice were purchased from National Laboratory Animal Center (Taipei, Taiwan) at 6–8 weeks old. Animals were housed in Animal Care Facility at Chang Gung Memorial Hospital (CGMH) under the standard guidelines from "Guide for the Care and Use of Laboratory Animals" and with the approval of CGMH Committee on Animal Research. Females were caged with males in the afternoon and checked for vaginal plugs the following morning. The day of the plug observed was designated as day 0 of the pregnancy.

### Mass culture of dispersed enterocytes

The gut was harvested from FVB/NCrl-Tg(Pgk1-EGFP)01Narl murine fetuses after cesarean delivery on gestational day 14. Fetal gut was treated with 1 mg/ml collagenase/dispase (Roche, Mannheim, Germany) in phosphate-buffered saline (PBS) for 15–30 min at 37 °C. Digested tissue was triturated and washed. Then, dispersed enterocytes at the dose of 1–2 × 10^6^ were seeded into tissue culture dishes (internal ∅87 mm TPP, 93,100) filled with self-renewal medium (SRM) [14–17]. SRM (100 ml) consisted of 50 ml low-glucose Dulbecco's modified Eagle medium, 30 ml neurobasal medium (Gibco), 15 ml chicken embryo extract, 100 µl retinoic acid (117 µM), 1 ml penicillin–streptomycin, 1 ml N_2_ supplement (Gibco), 2 ml B27 supplement (Gibco), 100 μl 2-mercaptoethanol (50 mM), basic fibroblast growth factor (20 ng/ml; Peprotech, London, UK) and insulin-like growth factor 1 (20 ng/ml; Peprotech). Cells were passaged every 4–5 days to lessen background mesenchymal cells. Then, enteric neurospheres were collected for transplantation.

### Flow cytometric analyses for enteric neurospheres [18]

Enteric neurospheres were dissociated by Accutase (STEMCELL technologies) and subjected to intracellular staining. After fixed, permeabilized and washed with BD Cytofix/Cytoperm™ Kit (BD Biosciences), cells were incubated with primary antibodies against p75 neurotrophin receptor (p75, ab8875, Abcam) and tubulin β3 (TUBB3, TUJ1, BioLegend), followed by fluorescence-conjugated secondary antibodies against the species the primary antibodies were raised in. After vigorous washes, cells were further treated with fluorescence-conjugated anti-S100 calcium-binding protein B (S100b, C-3, Santa Cruz Biotechnology). Finally, cells were acquired by BD FACSCantoTM II and analyzed with BD FACSDiva software.

### Ex vivo migration of neurosphere cells on gut explants

Adult murine colon of 2–3 cm was harvested, longitudinally cut open at the mesenteric side and washed with povidone-iodine and saline. Specimens were flattened with mucosa-side up on coverslips in the culture dish and mechanically denuded of mucosa by gentle curettages to expose muscularis. Adult gut explants were irrigated with PBS and finally rinsed with SRM. Fetal gut was obtained from gestational day 14 murine fetuses and cut into pieces of ≤ 5 mm in length on coverslips in the dish. Green fluorescence protein-positive (GFP^+^) neurospheres were implanted on the denuded muscularis surface of adult gut explant or at one end of fetal gut explant. The dishes were kept stationary for a few minutes to allow neurosphere attachment and then gently filled with SRM to flood the sample slides. The organotypic culture was held at 37 °C in a humidified, 5% CO_2_-containing incubator with SRM replacement every 3–4 days.

### Trans-anal ENSC transplantation

Under anesthesia, the anorectum of adult FVB/N mouse (5 for each gender, 8–12 weeks old) was disinfected using povidone-iodine. Recipient’s rectal mucosa was transfixed by three stay sutures of 6–0 PDS (taper point needle, ETHICON) near anorectal junction at 2, 6 and 10 o’clock positions and then artificially prolapsed by gentle traction of stay sutures. Neurospheres dissociated by Accutase were circumferentially injected into rectal submucosa at a dose of 1–2.5 × 10^6^ [1] in 50 μl saline (15–20 μl in each region), using an Ultra-Fine II insulin syringe, 31-G short needle (Becton, Dickinson and Company). Prolapsed rectal mucosa spontaneously returned after removal of stay sutures. After transplantation, mice were kept on warm blanket till waking up.

### Immunohistochemical staining of colon tissue sections for donor ENSC engraftment

The harvested colon specimens of recipients were fixed in 4% paraformaldehyde overnight and embedded in paraffin. Tissue sections were deparaffinized, rehydrated and then subjected to heat-induced antigen retrieval. Endogenous peroxidase was eliminated by hydrogen peroxide block (Thermo Fisher Scientific) for 15 min. The sections were permeabilized with Tween-20 and then blocked with 1% BSA. For immunohistochemical staining, tissue sections were first treated with rabbit polyclonal anti-GFP primary antibody (1:200, Cat No. GTX113617, GeneTex) for 1.5 h, followed by biotinylated goat anti-rabbit (rabbit specific HRP/DAB (ABC) detection IHC kit, Abcam) secondary antibody at room temperature for 10 min and then streptavidin-HRP in PBS for 10 min. Color was developed by adding chromogen substrate of DAB solution for 15 min. Finally, the sections were counterstained with hematoxylin for 5 min. The sample slides of the entire colon fixed in a Swiss roll fashion were specifically scanned and converted into high-definition digital data by NanoZoomer 2.0-RS. Images were analyzed by NDP.view2 viewing software for the histological locations of donor cell engraftment.

### Chimerism levels of donor ganglia within myenteric plexuses of recipients’ colorectum

The ganglia of myenteric plexuses in immunohistochemically-stained colorectum were enumerated under a 20 × objective. The levels of donor ganglion chimerism were determined by the number of GFP^+^ ganglia divided by the total ganglion number within the myenteric plexuses of the colorectum specimens.

### Immunofluorescence staining of colon tissue sections for enteric ganglia

Formalin-fixed, paraffin-embedded tissue sections were subjected to deparaffinization, rehydration, antigen retrieval and permeabilization as described above. Samples were first incubated with primary antibodies against GFP, followed by fluorescence-conjugated donkey anti-rabbit IgG (Poly4064, BioLegend), and finally treated with anti-TUBB3 and anti-glial fibrillary acidic protein (GFAP, 2E1.E9, BioLegend) antibodies directly conjugated with fluorescence. Visualization of the nuclei was achieved by Hoechst 33,342 staining (1: 20,000, Invitrogen). Sections were mounted with Dako fluorescence mounting medium. Images were taken using a Leica TCS SP8X confocal microscope.

### Statistical analyses

All numerical data were shown in boxplots. The equality of means was examined by one-way analysis of variance (ANOVA) among three groups with post hoc Fisher’s least significant difference (LSD) multiple comparisons. Differences were regarded as statistically significant at *p* < 0.05.

## Result

**Fig. 1:**
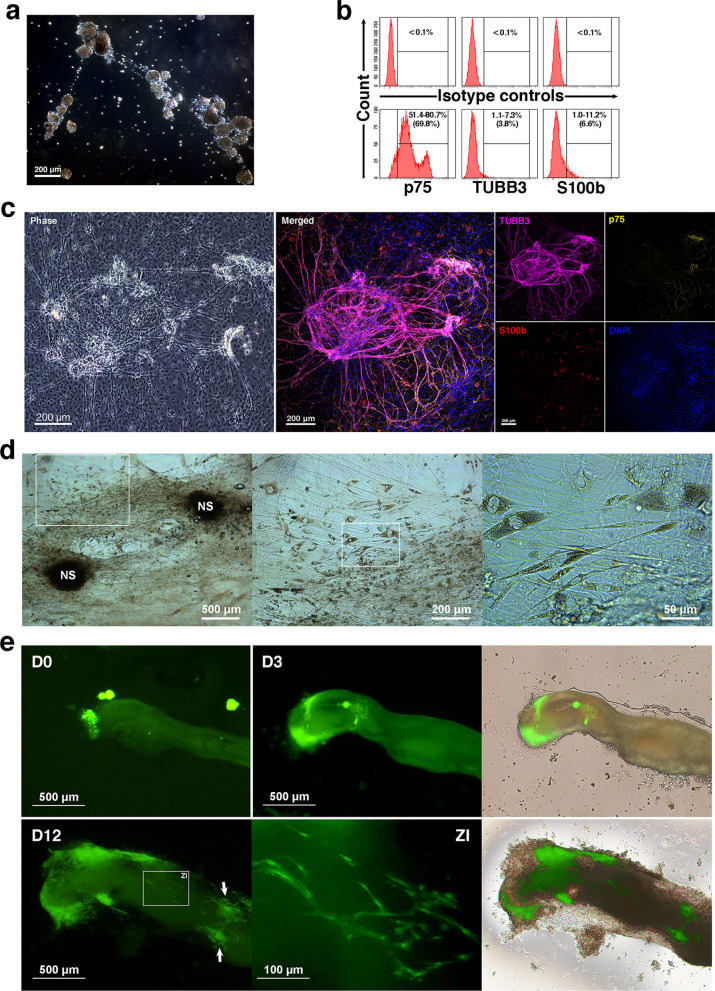
Characterization of enteric neurospheres and their migration ex vivo (**a**) Enteric neurospheres were generated after the 3rd cell passage in a representative mass culture of dispersed enterocytes. (**b**) Dissociated neurosphere cells were subjected to intracellular staining for p75 (ENSCs), TUBB3 (neurons) and S100b (gliocytes). Flow cytometric analyses revealed p75^+^ ENSCs of 51.4–80.7%, TUBB3^+^ neurons of 1.1–7.3% and S100b^+^ gliocytes of 1.0–11.2% (*n* = 10, the means shown in parentheses, lower panels). Their individual isotype controls are showed in the upper panels of the corresponding columns. (**c**) Enteric neurospheres were grown on fibronectin-coated coverslips flooded in SRM. Neurosphere cells migrated radially outwards to assemble neural networks. A representative image of enteric neural networks was taken live on day 14 (left panel). Then, the coverslip was subjected to immunofluorescence staining (right panels). The networks consisted of numerous ganglia, interconnected by neurite bundles. Ganglia harbored p75^+^ ENSCs, TUBB3^+^ neurons and S100b^+^ gliocytes. (**d**) Cells from neurospheres (NS) underwent migration on the muscularis of mucosa-denuded adult gut explants. Migratory cells exhibited polymorphism. Live cell images were captured without any prior staining under a phase-contrast microscope one week after neurosphere seeding, and zoomed in sequentially at selected areas. (**e**) GFP^+^ neurospheres were seeded at one end of fetal gut explants, counted as day 0 (D0). By D3, GFP^+^ cells had incorporated into the fetal gut, and migrated along its long axis. On D12, migratory neurosphere cells (ZI) contributed to the development of two network-like aggregates (arrows) far away from the seeding site

Enteric neurospheres (Fig. 1a) enriched from fetal gut were analyzed for ENSCs (p75^+^), neurons (TUBB3^+^) and gliocytes (S100b^+^) by flow cytometry after intracellular staining. Pooled neurospheres contained ENSCs of 54.1–80.7%, neurons of 1.1–7.3% and gliocytes of 1.0–11.2% (Fig. 1b). Cells from enteric neurospheres seeded on fibronectin-coated coverslips could migrate outwards to assemble neural networks that comprised ENSCs, neurons and gliocytes (Fig. 1c). They also exhibited migratory capacity ex vivo on adult (Fig. 1d) and fetal (Fig. 1e) gut explants.

**Fig. 2:**
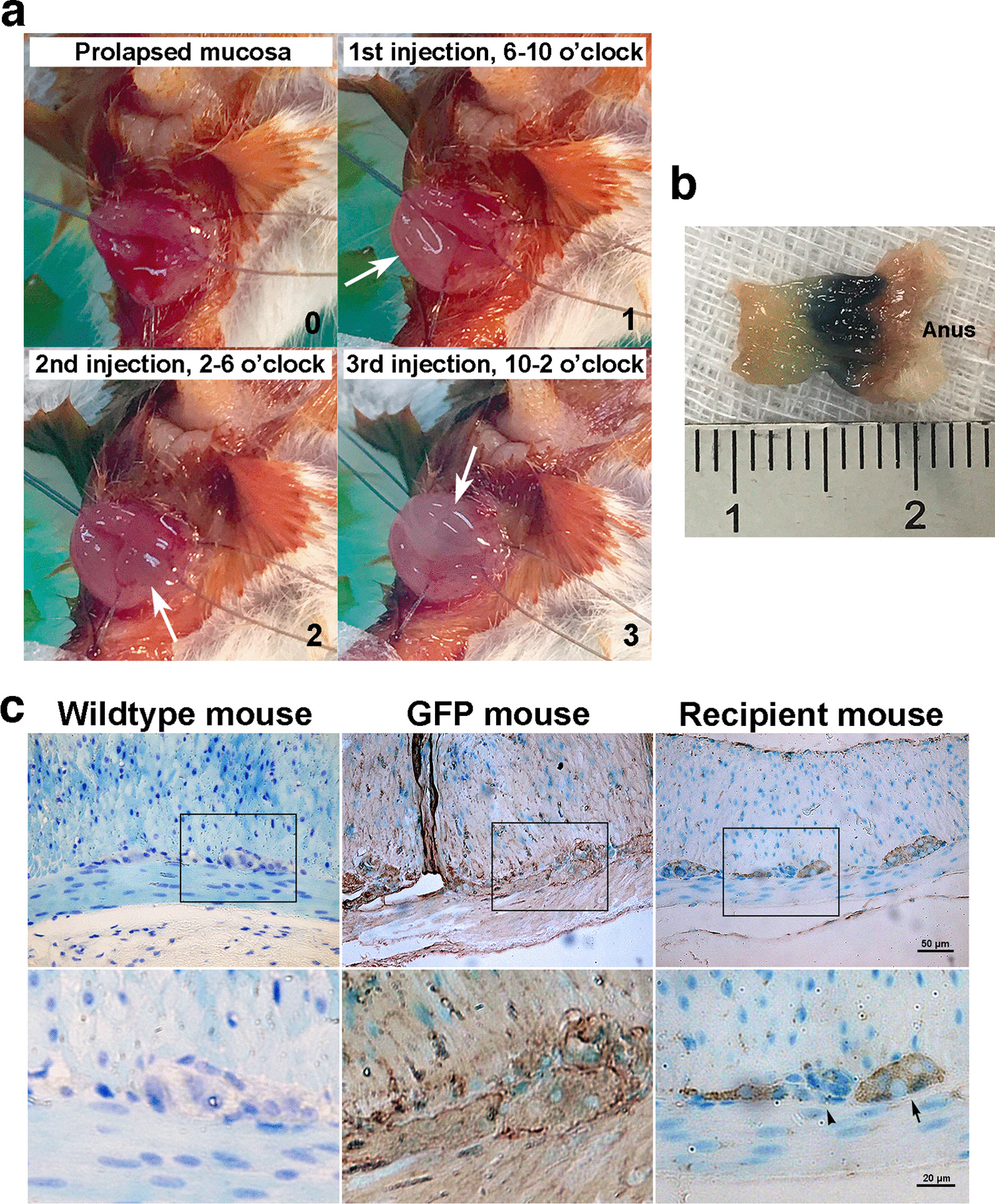
Trans-anal ENSC transplantation into rectal submucosa (**a**) Gentle traction of stay sutures artificially created a state of prolapsed rectal mucosa (0). It permitted precise rectal submucosal ENSC administration, leading to the swelling (arrows) of rectal mucosa (1–3). (**b**) This technique was further assessed, using the inocula mixed with methylene blue. The blue-stained anorectum, harvested and cut open longitudinally immediately after injection, indicated the proper inoculum delivery with an instant spreading of about 3–4 mm terminal rectum. Specimens shortened after harvest and fixation in formalin. (**c**) Four days after ENSC transplantation, a representative recipient’s rectum was subjected to immunohistochemical staining with anti-GFP antibodies. It demonstrated the proper intermuscular colonization of donor neurosphere cells (GFP^+^, arrows, right panel). There were endogenous ganglion cells (arrowhead). Positive (middle panel) and negative (left panel) controls were the rectum specimens from GFP^+^ and wildtype FVB/N mice, respectively

Dissociated GFP^+^ neurosphere cells were circumferentially injected into rectal submucosa after artificial prolapse of rectal mucosa (Fig. 2a and Additional file 1: Movie 1). Ten wildtype FVB/N recipients underwent rectal ENSC transplantation, and all survived the procedure. Immediately after injection, the inocula spread within terminal rectum of about 3–4 mm (Fig. 2b). Four days after transplantation, two recipients were killed for harvesting rectums. The specimens were subjected to immunohistochemical staining for GFP^+^ neurosphere cells. It showed that donor ENSCs or their progenies had the capacity to repopulate intermuscular area of the recipient’s rectum (Fig. 2c).

**Fig. 3:**
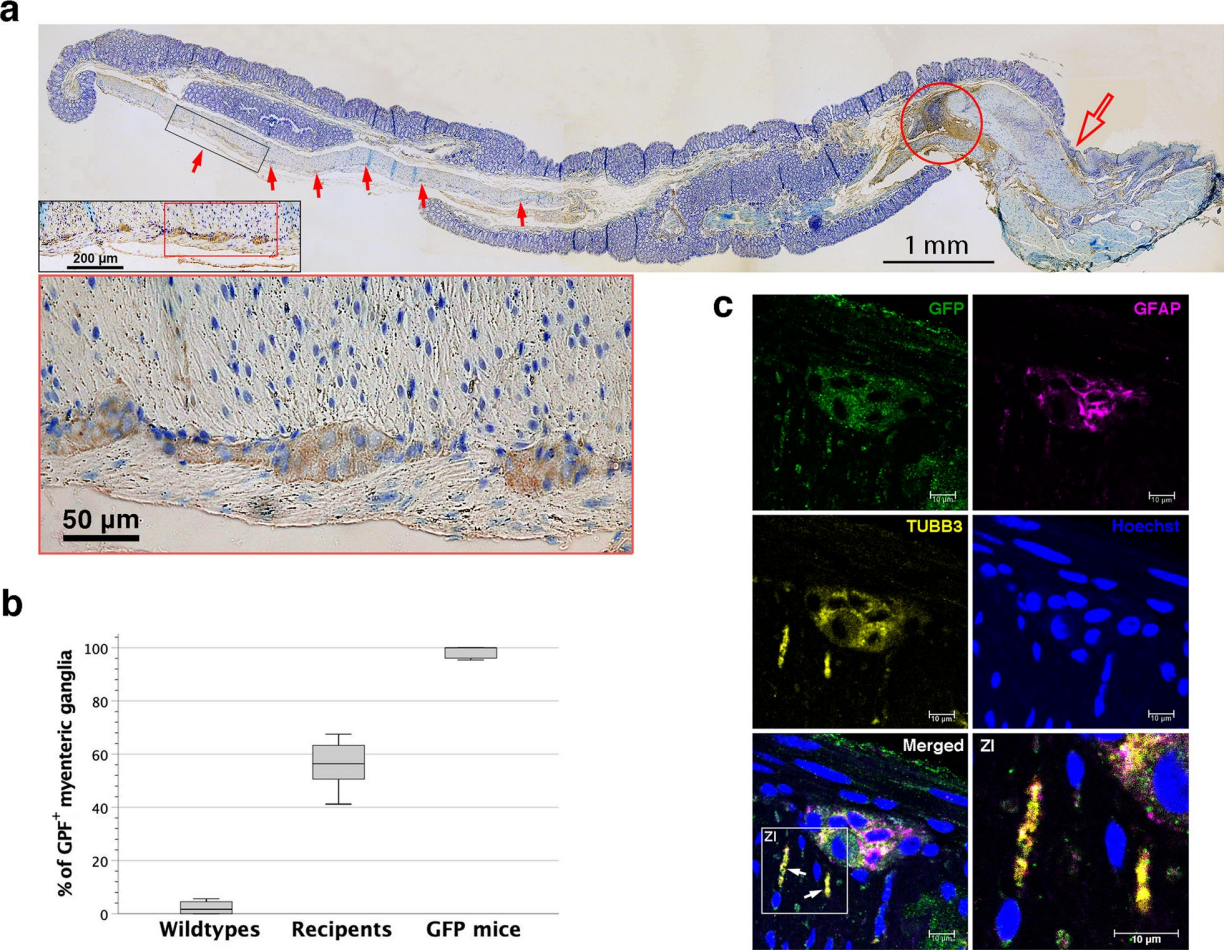
ENSC migration within neighboring colorectum one week after transplantation (**a**) Photomicrographs were taken through a microscope for the colorectum (distal one-third colon) after immunohistochemical staining and stitched together to create a panoramic image. The injection site was stained heavily (red circle). The pectinate line (open arrow) represented the transition from the simple columnar epithelium of the colon to the stratified squamous epithelium of anal skin. Donor cells (arrows) migrated rostrally to repopulate the intermuscular space of the recipient’s colon. An inset in the left lower corner showed a zoomed-in view of the intermuscular donor cell engraftment. (**b**) There was a mixed chimeric state of myenteric plexuses, containing donor-origin ganglia of 41.2–67.5%. It differed significantly from wildtype and GFP^+^ mice (*p* < 0.001, ANOVA, *n* = 6 in each group). (**c**) Immunofluorescence confocal microscopy disclosed that a representative myenteric ganglion contained enteric neurons and gliocytes of donor origin. The neurites outgrowing in the circular muscle layers were of donor-origin (arrows), as shown in zoomed-in view of boxed area (ZI)

Six recipient mice were killed 7–8 days after ENSC transplantation to harvest their distal third colon (2.5–3 cm). After immunohistochemical and immunofluorescence staining, colon specimens were examined whether transplanted cells could undergo intramural migration to reconstitute ganglionated plexuses. GFP^+^ cells were found to intermuscularly colonize the distal third colon (Fig. 3a), contributing to a chimeric state of myenteric plexuses with donor-origin ganglia of 41.2–67.5% (Fig. 3b). Immunofluorescence confocal microscopy further demonstrated that donor-origin enteric neurons and gliocytes assembled myenteric ganglia with neurite extension in muscularis (Figs. 3c). Thus, transplanted ENSCs or their progenies had the capacity to antiperistaltically reconstitute myenteric plexuses of the colon in the vicinity of the injection site.

**Fig. 4:**
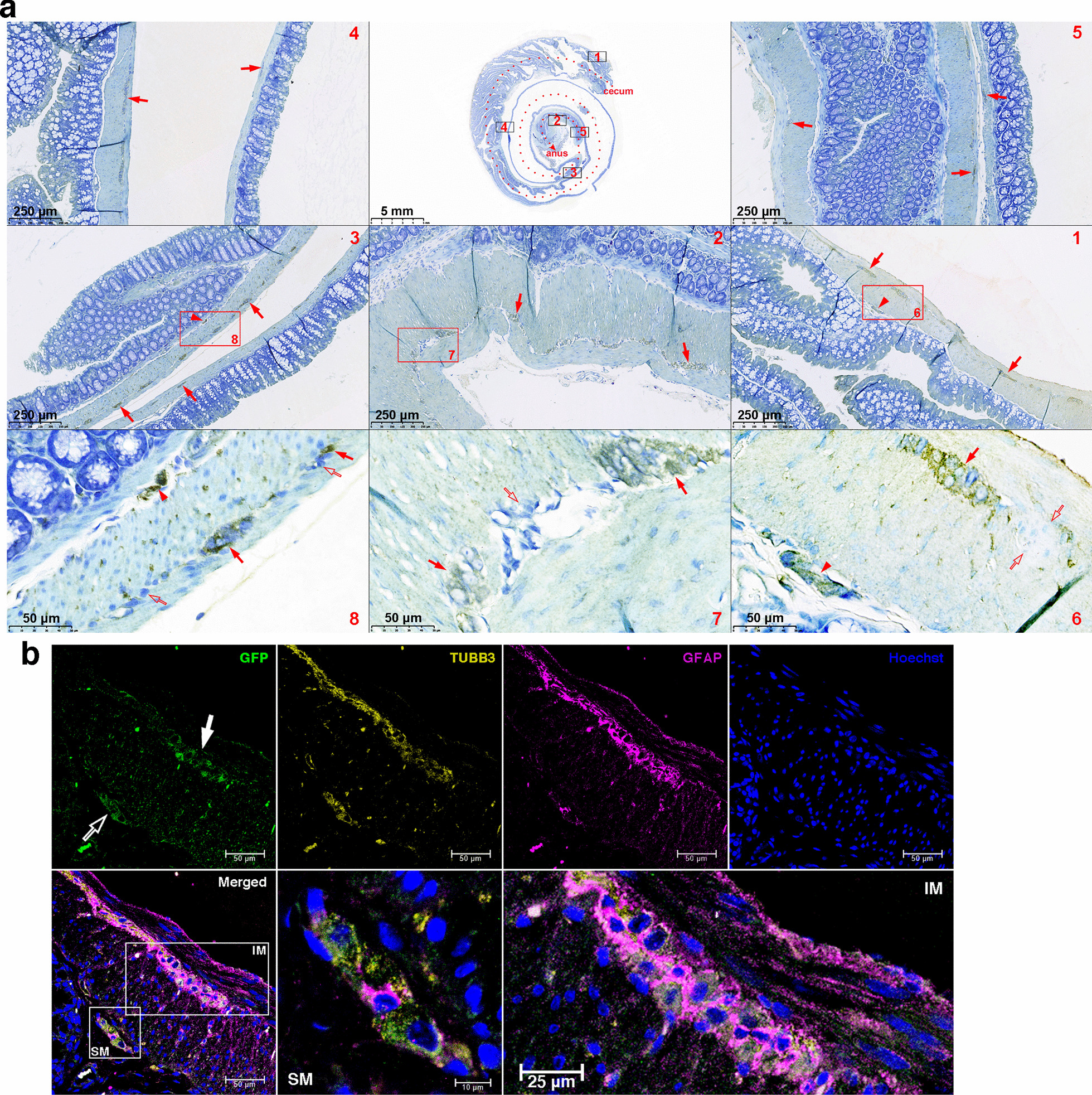
Long-range and long-term ganglion reconstitution (**a**) The entire colon of a representative recipient was fixed in a Swiss roll fashion and examined for donor cell engraftment by immunohistochemical staining two months after transplantation. The dotted line in red indicated the colonic lumen (upper middle panel). In panels 1–5 of the magnified views, donor cells mainly repopulated the intermuscular stratum (arrows) of the entire colon and sporadically inhabited the submucosal area (arrowhead). Boxed areas in panels 1–3 were further magnified (panels 6–8), showing the donor-origin myenteric (arrows) and submucosal (arrowhead) ganglion cells. There were also endogenous myenteric ganglion cells (blank arrows). (**b**) Immunofluorescence confocal microscopy confirmed myenteric (arrow) and submucosal (blank arrow) ganglion reconstitution by donor-origin neurons and gliocytes. Boxed areas were magnified to show submucosal (SM) and intermuscular (IM) donor-origin ganglia more in detail. Images were taken at the proximal colon and representative of 2 recipients with long-range and long-term ganglion reconstitution by donor ENSCs

In two recipients, their entire colons were obtained 2 months after transplantation to examine whether ENSCs could migrate long distances and reconstitute ENS plexuses long term. The colon fixed in the form of a Swiss roll was stained immunohistochemically. It showed that donor cells resided mostly in intermuscular area and occasionally in submucosa along the entire length of recipients’ colon (Fig. 4a). Immunofluorescence confocal microscopy confirmed that donor neurons and gliocytes assembled myenteric and even submucosal plexuses (Fig. 4b). These results indicated that ENSCs or their progenies transplanted into rectal submucosa could undergo long-distance caudorostral migration almost up to the cecum and reconstitute the ganglia in myenteric and submucosal plexuses long term.

## Discussion:

There have been several strategies for the introduction of ENSC inocula, either cell suspension or neurospheres, into the gut wall of laboratory animals [6], including serosal surface implantation [19], rectal muscle wall implantation via perianal incision [3], seromuscular [1, 10, 12, 13], intraperitoneal [20] and endoscopic injection [9]. Direct seromuscular injection after laparotomy represented the most popular but invasive route to deliver ENSCs. It allowed precise targeting to diseased bowel segments and multi-location injections, but had a limited capacity to deliver large-scale ENSC inocula for each single injection in mice [2, 10, 21] except for the use of Matrigel as carriers to prevent backflow leakage after injection [1, 11]. When combined with laparoscopic techniques, trans-serosal injection becomes minimally invasive and poses the great potential for clinical application. However, undefined cell spreading remained a major concern after a focal ENSC seromuscular injection except that human ENS precursors injected into postnatal murine cecal wall could rostrocaudally migrate long distances [1]. Whether homologous ENSCs have controllable migration patterns following focal seromuscular injection or spread randomly remains unknown, calling the reproducibility of site-specific injections into question [6]. Thus, cell distribution after focal seromuscular injection demands further specifications to ensure the optimal injection sites of a diseased bowel segment and the predictable outcome.

It was reported that ENSC inocula could be delivered intraluminally into bowel wall via endoscopy [9]. Transplanted cells spread circumferentially within the submucosal plexus following injection and migrated longitudinally for around 1 mm within one week. Endoscopic approach involved technical difficulty and the risk of intestinal perforation. Accurate localization of the lesions for endoscopic injection might pose an additional challenge. Intraperitoneal ENSC injection was relatively noninvasive, but ENSCs preferentially colonized small rather than large bowel [20]. Thus, intraperitoneal ENSC injection lacks the controllable or predictable targeting at the diseased gut site, raising doubts about its clinical applicability. ENSCs mixed in biodegradable fibrin glue had been implanted onto the serosal surface of murine intestine [19]. Although implanted cells could migrate downwards focally to repopulate the longitudinal muscle layers, only few cells passed through the longitudinal muscle to reach myenteric plexuses.

For the first time ever, ENSC transplantation was conducted via trans-anal approach. ENSCs introduced into rectal submucosa migrated intramurally in the rostral direction. They differentiated toward TUBB3^+^ neurons and GFAP^+^ gliocytes to reconstitute myenteric or submucosal plexuses of the neighboring colorectum within 1 week, and even the entire colon by 2 months after transplantation. However, it necessitates a larger number of animal subjects to further explicate the chance that ENSCs can achieve long-distance migration, and the relationship of cell doses to the migration distance and even the migration speed of ENSCs in postnatal colonic milieu. Notably, ENSC transplantation also led to the engraftment of GFP^+^ cells that were negative for TUBB3 and GFAP (Figs. 3c and 4b) within recipients’ ganglia or muscularis. They might be undifferentiated ENSCs or myofibroblasts as ENSCs possessed the potential to differentiate toward myofibroblasts [15, 22]. The histologically stratum-accurate ganglion reconstitution by donor ENSCs within the recipient gut wall justified the feasibility of rectal ENSC inoculation to replace damaged neurons in Chagas disease or even replenish missing neurons in HSCR [6]. In about 75–80% HSCR patients, aganglionosis was confined to rectosigmoid. Thus, rectal submucosal injection would be the most scientifically sound approach to ENSC therapy for short-segment HSCR, and even long-segment HSCR given ENSCs’ ability to antiperistaltically migrate long distances along the entire colon.

Although aganglionic colon was reportedly permissive to the migration and differentiation of ENSCs implanted in rectal muscle layer via perianal incision [3], ENSC spreading after rectal injection demands further evaluations in aganglionic gut caused by repulsive effects of abnormal gut extracellular matrix molecules on ENSC migration [23, 24]. Notably, rectal ENSC injection caused dominant myenteric ganglion reconstitution, in sharp contrast to a preference for submucosal ganglion reconstitution of the entire colon following cecal seromuscular injection of ENS precursors derived from human pluripotent stem cells [1]. The distinction in ganglion reconstitution was not necessarily relevant to cell administration route (rectal vs. cecal approach), but had to take into consideration the difference in transplantation model (syngeneic vs. xerogeneic) and stem cell origin (gut-derived vs. embryonic stem cell derived). Although ENSC engraftment after direct seromuscular injection proved the generation of functional enteric neurons [1, 10, 12, 25], ganglion reconstitution after rectal ENSC transplantation demands further functional assays in the future to address functional innervation or motility restoration of diseased gut and substantiate its therapeutic significance for enteric neuropathy.

## Conclusion

Trans-anal rectal ENSC injection was far less invasive as opposed to direct seromuscular injection via laparotomy or laparoscopy. Transplanted ENSCs or their progenies might undergo caudorostral migration in postnatal colonic milieu to reconstitute submucosal and myenteric ganglia long-term and long-distance along the entire length of the colon. Not only does this proof-of-principle study open up the new approach to ENSC delivery in laboratory animals, but also casts light on the feasibility of replacing damaged or replenishing missing enteric neurons by minimally invasive rectal ENSC transplantation.

## Supplementary Information

**Additional file 1**: Movie 1. Rectal submucosal injection of ENSC inocula in mice. Murine rectal mucosa was prolapsed by 3 stay sutures of 6-0 PDS at 2, 6 and 10 o’clock of anorectal junction. The movie showed the process of rectal submucosal injection using a 31G short needle Ultra-Fine II insulin syringe. Successful ENSC inoculation into rectal submucosa gave rise to immediate swelling of rectal mucosa.

## Data Availability

All data generated or analyzed during this study are included in this published article.

## Abbreviations

ENSCs: Enteric neural stem cells
ENS: Enteric nervous system
GFAP: Glial fibrillary acidic protein
GFP: Green fluorescence protein
HSCR: Hirschsprung’s disease
p75: p75 neurotrophin receptor
TUBB3: Tubulin β3
S100b: S100 calcium-binding protein B
SRM: Self-renewal medium
